# Dietary Patterns in Primary School are of Prospective Relevance for the Development of Body Composition in Two German Pediatric Populations

**DOI:** 10.3390/nu10101442

**Published:** 2018-10-05

**Authors:** Maike Wolters, Gesa Joslowski, Sandra Plachta-Danielzik, Marie Standl, Manfred J. Müller, Wolfgang Ahrens, Anette E. Buyken

**Affiliations:** 1 Leibniz Institute for Prevention Research and Epidemiology—BIPS, Department: Epidemiological Methods and Etiologic Research, Achterstr. 30, 28359 Bremen, Germany; ahrens@leibniz-bips.de; 2 IEL—Nutritional Epidemiology, University of Bonn, DONALD Study, Heinstück 11, 44225 Dortmund, Germany; gesa.joslowski@gmx.de (G.J.); anette.buyken@uni-paderborn.de (A.E.B.); 3 Institute of Human Nutrition and Food Science, Christian-Albrechts University, 24118 Kiel, Germany; s.plachta-danielzik@kompetenznetz-ced.de (S.P.-D.); mmueller@nutrfoodsc.uni-kiel.de (M.J.M.); 4 Institute of Epidemiology I, Helmholtz Zentrum München—German Research Center for Environmental Health, D-85764 Neuherberg, Germany; marie.standl@helmholtz-muenchen.de; 5 Institute of Nutrition, Consumption and Health, Faculty of Natural Science, University Paderborn, 33098 Paderborn, Germany

**Keywords:** body composition, primary school, dietary pattern, principal component analysis, reduced rank regression, prevention

## Abstract

This study performed comparative analyses in two pediatric cohorts to identify dietary patterns during primary school years and examined their relevance to body composition development. Nutritional and anthropometric data at the beginning of primary school and two or four years later were available from 298 and 372 participants of IDEFICS-Germany (Identification and prevention of Dietary-induced and lifestyle-induced health Effects In Children and infants Study) and the KOPS (Kiel Obesity Prevention Study) cohort, respectively. Principal component analyses (PCA) and reduced rank regression (RRR) were used to identify dietary patterns at baseline and patterns of change in food group intake during primary school years. RRR extracted patterns explaining variations in changes in body mass index (BMI), fat mass index (FMI), and waist-to-height-ratio (WtHR). Associations between pattern adherence and excess gain in BMI, FMI, or WtHR (>75th percentile) during primary school years were examined using logistic regression. Among PCA patterns, only a change towards a more Mediterranean food choice during primary school years were associated with a favorable body composition development in IDEFICS-Germany (*p* < 0.05). In KOPS, RRR patterns characterized by a frequent consumption of fast foods or starchy carbohydrate foods were consistently associated with an excess gain in BMI and WtHR (all *p* < 0.005). In IDEFICS-Germany, excess gain in BMI, FMI, and WtHR were predicted by a frequent consumption of nuts, meat, and pizza at baseline and a decrease in the consumption frequency of protein sources and snack carbohydrates during primary school years (all *p* < 0.01). The study confirms an adverse impact of fast food consumption on body composition during primary school years. Combinations of protein and carbohydrate sources deserve further investigation.

## 1. Introduction

Most Western societies report the highest prevalence of overweight/obesity among children and adolescents at the end of primary school [1,2]. Thus, primary school years are now regarded as a further window of opportunity when interventions targeting development or the reversal of becoming overweight or obese are particularly warranted [3,4,5,6,7,8].

Upon entry into primary school, many aspects of a child’s daily routine change, which may all contribute to the lower remission of overweight during primary school years as compared to pre-school years. Importantly, nutritional behavior may change substantially and these changes may best be captured by dietary pattern analysis, which allows insights into the joint relevance of multiple dietary components for overweight development. Yet, to date, few studies examined the prospective relevance of dietary patterns for the development of body composition [9,10,11,12,13,14,15] and most of these were performed among adolescents [10,11,13]. In addition, such analyses should often consider BMI only [10,11,12], which is, however, only a proxy measure of adiposity. Consideration of more adiposity-specific measures such as fat mass or waist circumference is, therefore, warranted [16,17,18,19].

Additionally, patterns of dietary change during primary school years may be of relevance for an unfavorable development of body composition during this period. In a previous analysis, we observed that both the selection of unfavorable carbohydrate sources (more white bread, less pulses, and whole grain bread) at the beginning of primary school and an increased consumption of processed savory food during primary school years were related to adverse changes in BMI and fat mass during primary school years in a sample of German school children participating in the DONALD Study (Dortmund Nutritional and Longitudinally Designed Study) [9]. These patterns were identified by using reduced rank regression (RRR) to identify dietary patterns best explaining variation in changes of body composition during primary school years. Ideally, these dietary patterns should be applied to another cohort so as to confirm their relevance for German pediatric populations in general. In practice, this is, however, not possible because of different dietary assessment methods used in different studies (e.g., German pediatric cohort studies often assess consumption frequency only, i.e., use food propensity questionnaires [20]). Comparative analysis based on a different dietary assessment method but using a similar analytic approach is, therefore, a feasible alternative approach. Similar dietary patterns identified with this analytical approach would be highly informative for the formulation of preventive and interventional strategies targeted at primary school age children.

Therefore, the aims of the present study were to identify and describe dietary patterns at the beginning of the primary school period as well as patterns of dietary changes during the course of primary school in two German pediatric cohort studies known as the Kiel Obesity Prevention Study (KOPS) and the Identification and prevention of Dietary-induced and lifestyle-induced health Effects In Children and infants (IDEFICS)-Germany cohort. In a further step, we illustrated the impact of adherence to the identified dietary patterns on excess gain in the body mass index (BMI), fat mass index (FMI), or waist-to-height ratio (WtHR) (increases >75th percentile) during primary school years.

## 2. Methods

### 2.1. Study Populations

The study designs of the KOPS and the IDEFICS-Germany study were described previously [16,21,22]. KOPS is a cohort study to investigate determinants and preventive measures of childhood obesity. It was performed between 1996 and 2001 in the context of a school entry health examination [23,24]. A representative group of 4997 children participated in the study [24,25]. At the end of the primary school period (fourth grade), follow-up information was collected from 1764 children (35% of the original population) during examinations performed in the school setting between 2000 and 2005 [23,24]. For the present analyses, complete information on dietary habits at baseline (age 5 to 7 years) and follow-up (age 9 to 11 years) was available for 415 children. Of these, 372 had complete anthropometric data at both time points as well as information on potential confounding factors.

The IDEFICS-Germany study is a multicenter study designed to investigate and prevent diet-related and lifestyle-related disorders among European children (aged 2–9.9 years at baseline). The present analysis is based on data of the German subsample. In 2007/2008, 2066 children were recruited from kindergartens and schools in Germany. Anthropometric measurements were performed at baseline and at the two-year follow-up and dietary and lifestyle questionnaires were administered. A total of 993 children had complete dietary data at baseline and follow-up. Of these, 312 children provided dietary data at the beginning of primary school (i.e., age 5 to 7 years) and at least two years later. Complete anthropometric measurements at baseline and follow-up and data on potential covariates were available for 298 children included in the analyses.

This study was conducted according to the guidelines laid down in the Declaration of Helsinki and all procedures involving human subjects were approved by the local ethics committee in Kiel and the ethics review board of the University of Bremen written informed consent was obtained from the parents or legal caregivers of all children. 

### 2.2. Nutritional Assessment

In KOPS, dietary intake was assessed using a 24 item food propensity questionnaire (FPQ) based on the WHO MONICA FFQ (food frequency questionnaire) adapted to children [26] (see Table S1). It was completed by the parents or primary caregiver who provided dietary information over the last six months. The FPQ—i.e., an FFQ inquiring consumption frequencies but not the amounts consumed was validated against a seven day diet record in 24 and 61 5–7 and 9–11 year old children, respectively [27]. Consumption frequencies were asked from mutually exclusive alternatives: never or less than once a week, 1 to 2 times per week, and 3 to 6 times per week or daily.

The Children’s Eating Habits Questionnaire (CEHQ) was used to assess dietary intake in the IDEFICS-Germany study [28]. This reproducible and validated instrument [28,29,30] inquiring the consumption frequency of 43 foods and beverages (see Table S2) was completed by parents or other caregivers. Frequencies of consumption during the past month were asked from mutually exclusive alternatives: never or less than once a week, 1 to 3 times per week, 4 to 6 times per week, once per day, twice per day, and three or more times per day.

### 2.3. Anthropometric Measurements and Calculations

Anthropometric measurements in KOPS were performed by trained nutritionists and physicians [25]. Weight and height were measured to the nearest of 0.1 kg and 0.5 cm with a calibrated electronic scale and a stadiometer (Seca, Hamburg, Germany). Waist circumference was measured midway between the lowest rib and the top of the iliac crest at the end of gentle expiration with an inelastic tape. A bioelectrical impedance analysis (BIA) was performed by using a terapolar bio-impedance analyzer (BIA 2000-C, Data Input, Frankfurt/M, Germany) and fat mass was calculated with an algorithm developed in Kiel using air-displacement plethysmography as the reference method [16,31].

In IDEFICS-Germany, anthropometric measurements included weight, height, BIA, and waist circumference. Children were barefoot and wore only underwear and a T-shirt. Weight measurements to the nearest 0.1 kg and BIA were carried out using an electronic scale (Tanita BC 420 SMA, Tanita Europe GmbH, Sindelfingen, Germany). Height was measured using a telescopic height measuring instrument (Seca 225 stadiometer, Hamburg, Germany) to the nearest 0.1 cm. Fat mass was calculated with an algorithm derived from BIA [32]. Waist circumference was measured using an inelastic Seca 200 tape (Seca, Hamburg, Germany) according to World Health Organization Standards [33].

For both study samples, BMI was calculated from measured height and weight (weight (kg)/height^2^ (m^2^)). FMI and FFMI were calculated relating fat mass and fat free mass (weight—fat mass) to height^2^ (m^2^). For WtHR (waist circumference(m)/height(cm), a simple practical non-invasive tool reflecting visceral fat of a value >0.5 was considered to indicate excessive upper body fat accumulation [34]. Since absolute measures are considered a better alternative than *z*-scores when assessing adiposity changes [35], the response variables were the changes in BMI, FMI, and WtHR between the end and the beginning of the primary school period. Being overweight was defined according to the International Obesity Task Force criteria [36]. 

### 2.4. Statistical Methods

To facilitate comparison to the findings from the DONALD study, the statistical approach in the present analysis was analogous to that in Diethelm et al. [9]. Dietary patterns at the beginning of the primary school period and patterns of changes during primary school years were derived by two different empirical methods: PCA and RRR. Intake frequencies were standardized by using *z*-scores (by age group and sex (mean = 0, SD = 1)). Due to the extracted dietary patterns being similar for boys and girls in both cohorts, the data were pooled for the analyses.

#### 2.4.1. PCA

PCA analyses were conducted by using the PROC FACTOR procedure in SAS. The first PCA was conducted to identify dietary patterns at the beginning of the primary school period (PCA baseline pattern) while the second PCA identified patterns of dietary changes between the end and the beginning of the primary school period (PCA change pattern). For this purpose, changes in the intake frequencies of food groups were obtained (standardized intake frequencies at the end of the primary school period minus standardized intake frequencies at the beginning of the primary school period). 

To select the baseline and change patterns to be retained in analyses, the following criteria were used: (1) eigenvalues exceeding 1 (based on the rationale that each component should explain more variance than a single variable in the data set), (2) the scree plot (a graphical presentation of the eigenvalues where a break indicates how many factors should be retained), and (3) factor interpretability [37,38]. Two baseline patterns and three change patterns were retained for further analyses. After a varimax rotation, food groups with factor loadings ≥ |0.4| were considered as contributing to a pattern. For each pattern, more than three food groups were retained, which is considered an adequate number for good interpretability [38]. According to the approach of simplified pattern variables, the individual PCA factor scores (resembling adherence to the respective pattern) were based solely on the sum of the unweighted standardized frequencies of food groups that were loaded high (i.e., ≥|0.4|) at the respective pattern [39].

#### 2.4.2. RRR

RRR analyses were used as a second approach. RRR is commonly applied to a set of intermediary response variables presumed to link diet to the health outcome [40]. Since current scientific evidence does not substantiate strong associations between specific nutrients and body composition development, RRR was used to directly extract dietary patterns best explaining the variation of changes in BMI, FMI, and WtHR during the primary school period; i.e., the method was used in a purely exploratory way.

RRR analyses were conducted via the PROC PLS procedure in SAS (option method = RRR) [40]. The RRR extracts linear combinations (i.e., RRR patterns) of predictor variables that explain as much variation in response variables as possible. For the first RRR, standardized intake frequencies of food groups at the beginning of the primary school period were used as predictor variables. The response variables were the changes in BMI, FMI, and WtHR between the end and the beginning of the primary school period in which each was adjusted for baseline BMI, FMI, and WtHR at the beginning of the primary school period, respectively, by applying the residual method [41] (RRR baseline factors). For the second RRR, changes in standardized intake frequencies of food groups between the end and the beginning of the primary school period were used as predictor variables. Changes in BMI, FMI, and WtHR between the end and the beginning of the primary school period adjusted for the baseline body composition were, again, the response variables (RRR change factors). 

The number of factors extracted by RRR from the predictor variables is always equal to the number of response variables, i.e., three factors were identified by each RRR. However, only those patterns explaining the largest amount of response variation (as indicated by a break) were retained for further analyses. As for PCA, the individual RRR factor scores were calculated, according to the simplified pattern approach [39] including all food groups with a factor loading ≥|0.2|.

#### 2.4.3. Logistic Regression Analyses

The PCA and RRR factors were used as independent predictors in the logistic regression models (PROC LOGISTIC) by estimating odds ratios (ORs) with 95% confidence intervals (95% CIs) of excess gain in BMI, FMI, or WtHR during primary school years with the lower three quartiles of increases in BMI, FMI, or WtHR serving as the reference category. 

By definition, RRR patterns will be predictive of changes in BMI, FMI, and WtHR during primary school years, i.e., the response variables for which they were derived. Thus, our main interest was to (i) illustrate the effect sizes of the predicted OR and (ii) to investigate whether the obtained pattern would still be predictive after adjustment for potentially confounding factors [9]. Adjusted OR for excess gain in BMI, FMI, and WtHR are categorized by tertiles of RRR or PCA factors scores of adherence. The *p*-values for a linear trend refer to logistic regression models with continuous pattern scores of adherence as the independent variable. Potentially confounding variables were initially examined separately and included in the fully adjusted model only if they modified regression coefficients of the pattern scores in the unadjusted models by ≥10%. To ensure comparability between the models, we included all variables that met this criterion in any of the models investigating the relationship between a dietary pattern (baseline or change patterns) and the outcomes (excess gain in BMI, FMI, or WtHR).

The following covariates inquired from the parents by self-administered questionnaires were considered as potentially confounding factors: child’s birth weight, breast feeding practice, smoking during pregnancy, parental education, parental weight and height, income, the child’s physical activity, and the migration background (IDEFICS only). 

Differences between baseline and follow-up were tested using the Wilcoxon rank sum test for continuous variables and the Chi-square test for categorical variables. SAS procedures (SAS, version 9.2, SAS Institute Inc., Cary, NC, USA) were used for data analysis. A *p*-value <0.05 was considered statistically significant.

## 3. Results

The characteristics of both study samples are given in Table 1. The anthropometric characteristics of the participants stratified by the beginning and the end of primary school years are summarized in Table 2. In the KOPS sample, waist circumference, BMI, and FFMI increased during primary school years while WtHR decreased and FMI did not change. Similarly, waist circumference, BMI, FMI, and FFMI increased during primary school years in the IDEFICS-Germany sample while WtHR decreased.

### 3.1. Dietary Patterns

For the KOPS sample, the dietary pattern obtained by PCA and RRR are presented in Table 3. The PCA baseline pattern 1 is characterized by a frequent consumption of ‘fast food’ (fish fingers, curry-sausage, or lasagna) while food groups characterizing PCA baseline pattern 2 could be summarized as ‘healthy’ (wholegrain, vegetables, and fruits). The PCA change pattern 1 reflects an increase in the consumption frequency of fast foods while the PCA change patterns 2 and 3 reflect changes in diet towards a healthier and an unhealthier dietary pattern, i.e., an ‘increase in the consumption of vegetables and fruits’ and a ‘change towards unhealthy carbohydrates’ (increase in white bread and savory bakery goods and decrease in whole-grain bread), respectively. The RRR patterns contained more disjoint food groups than the PCA patterns. The RRR baseline pattern was labelled a ‘fast food pattern’ characterized by a frequent intake of lemonade, children’s yogurt, and a seldom intake of whole-grain bread, cheese, curd, and yogurt. The RRR change pattern increase in frequency of fast foods and starchy carbohydrate foods was characterized by an increase in the consumption frequency of carbohydrate sources such as whole-grain bread, potatoes, and pizza as well as fish sticks while the consumption frequency of vegetables decreased.

For the IDEFICS-Germany sample, dietary patterns obtained by PCA and RRR are given in Table 4. The PCA baseline pattern 1 termed ‘snack pattern’ was characterized by the frequent consumption of snack foods (sweet snacks, fried potatoes, croquettes, ketchup, savory snacks, and sweetened drinks) while frequent consumption of plain unsweetened yogurt or kefir, dishes of milled cereals, nuts, and pizza dominate the PCA baseline pattern 2 (‘Mediterranean type pattern’). The PCA change pattern 1 termed ‘change towards a Mediterranean type pattern’ is dominated by increases in the consumption frequency of nuts, pasta, fresh meat, and pizza. Increases in the consumption frequency of cooked vegetables, potatoes, beans, and legumes but also sweetened drinks, butter/ margarine, and fresh fruits characterize the PCA change pattern 2 (‘change towards a traditional type pattern’) while the PCA change pattern 3 (‘change towards a snack pattern’) is dominated by increases in the consumption frequency of sweet snacks such as biscuits or pastries, candies, ice creams, and savory snack foods such as crisps or popcorn. The RRR baseline pattern was labeled ‘Nuts, meat, and pizza pattern’ because it is characterized by frequent intakes of nuts, fresh meats, pizza but also yogurt, jam, and honey as well as a seldom consumption of cooked vegetables, potatoes, beans, legumes, and sweetened breakfast cereals. The RRR change pattern, ‘decrease in the consumption of animal protein sources and snack carbohydrates’, was characterized by an increase in the consumption of ’reduced-fat products on bread’ and decreases in the consumption frequency of fresh meat, savory pastries, fried or scrambled eggs, sweetened drinks, nuts, seeds, and dried fruits.

### 3.2. Logistic Regression Analyses

In KOPS, no significant association was found between PCA baseline patterns (‘fast food pattern’ and ‘wholegrain, vegetables & fruits’) and excess gain in BMI, FMI, or WtHR during the primary school period (Table 5). Among the PCA change patterns, only the adherence to an ‘increase in the consumption of fast food’ was significantly associated with higher odds for an excess gain in FMI after adjustment for parental overweight, parental education, and physical activity (*p*_trend_ = 0.0411). However, no similar associations were seen with odds for an excess gain in BMI or WtHR.

**Figure 1:**
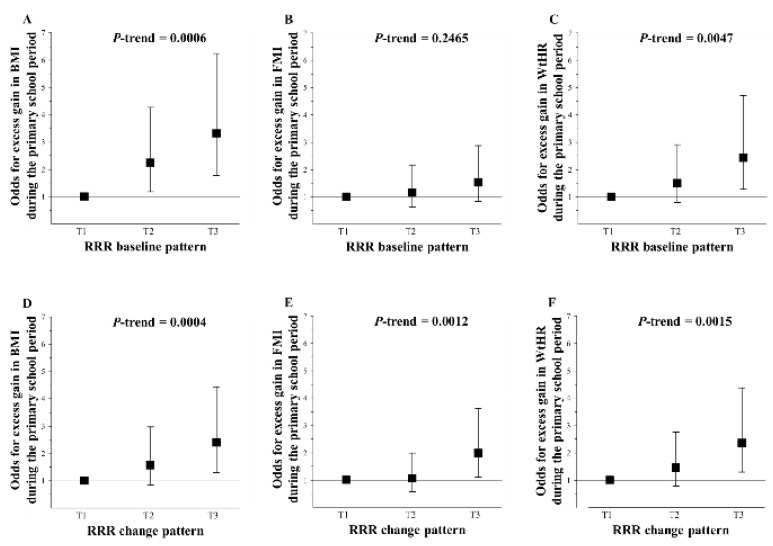
Odds for excess gains in BMI (panel **A** and **D**), fat-mass index (FMI, panel **B** and **E**), and waist-to-height ratio (WtHR, panel **C** and **F** during the primary school period according to tertiles of adherence to RRR baseline and change patterns (‘fast food pattern’ and ‘increase in the consumption of fast foods and starchy carbohydrate foods’, respectively)–KOPS sample (*n* = 372). Values are odd ratios (95% confidence intervals) presented in tertiles of adherence to the respective dietary pattern. Changes in body composition were adjusted for baseline body composition using the residual method. For the logistic regression analyses, the outcomes were excess gains in BMI, FMI, or WtHR defined as gains >75th percentile, adjusted for parental overweight (BMI ≥ 25 kg/m^2^, yes/no), parental education (≥12 years of schooling, yes/no), and physical activity (very low, low, middle, and high). The *p*-values are based on logistic regression models with dietary pattern scores as a continuous variable. T, Tertile.

Figure 1 shows that a closer adherence to the RRR baseline pattern (‘fast food pattern’) was independently related to higher odds for an excess gain in BMI (Panel A, *p*_trend_ = 0.0006) and WtHR (Panel C, *p*_trend_ = 0.0047) but not in FMI (Panel B, *p*_trend_ = 0.2465) during primary school in the KOPS sample. Similarly, a closer adherence to the RRR change pattern (‘increase in frequency of fast foods and starchy carbohydrate foods’) predicted excess gain in BMI, FMI, and WtHR during the primary school period (Panel D–F, all *p*_trend_ < 0.002). Overall, those in the highest tertile of adherence were at least two times more likely to have excessively gained than their counterparts in the lowest tertile of adherence.

In IDEFICS-Germany, higher adherence to the PCA change pattern (‘change towards a Mediterranean type pattern was associated with lower odds for excess gain in FMI, WtHR, and BMI (*p* < 0.05) (Table 6). All other PCA baseline and change patterns were not associated with changes in body composition during primary school years.

**Figure 2:**
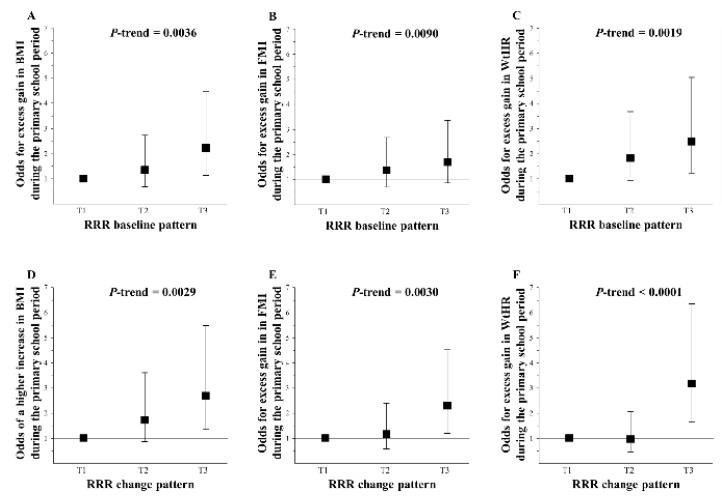
Odds for excess gains in BMI (panel **A** and **D**), fat-mass index (FMI, panel **B** and **E**), and waist-to-height ratio (WtHR, panel **C** and **F** during the primary school period according to tertiles of adherence to the RRR baseline and change patterns (‘Nuts, meat, and pizza pattern’ and ‘decrease in the consumption of protein sources and snack carbohydrates’, respectively)—IDEFICS-Germany sample (*n* = 298). Values are odd ratios (95% confidence intervals) presented in tertiles of the respective dietary pattern. Changes in body composition were adjusted for baseline body composition (BMI, FMI, or WtHR, respectively) using the residual method. For the logistic regression analyses, the outcomes were excess gains in BMI, FMI, or WtHR, which were defined as gains >75th percentile, adjusted for parental overweight (BMI ≥ 25 kg/m^2^; yes/no), smoking during pregnancy (yes/no), and migration background (born in Germany, yes/no). Models for BMI and FMI also adjust for low income (<1.100 € per month, (no/yes/unknown): 8% of values were inputted using the missing indicator method resulting in a coding of 0, 1, and 2, respectively [42]), *p*-values based on multiple logistic regression models with dietary pattern scores as continuous variables. T, Tertile.

Figure 2 illustrates that both the RRR baseline and change patterns remained independently associated with substantially increased odds for excess gains in BMI, FMI, and WtHR during the primary school years in the IDEFICS-Germany sample after an adjustment for potential confounders (all *p*_trend_ < 0.01).

## 4. Discussion

The present study provides new prospective evidence from two pediatric samples suggesting a relevance of dietary patterns during the primary school period identified by RRR for the development of body composition in primary school years. Our data support a potentially detrimental role of dietary patterns characterized by fast foods for BMI, FMI, and WtHR development during this critical time period. On a more exploratory level, this study also suggests that future research should shed light on the relevance of combinations of protein and carbohydrate sources for body composition development in school children.

Both PCA and RRR identified dietary patterns characterized by a preferred consumption of fast foods, which were associated with excess gains in adiposity measures both prospectively and concurrently in the KOPS cohort. Our findings are broadly in line with the ALSPAC study, which reported that an energy-dense, low-fiber, high-fat RRR dietary pattern at 7 years of age but not at 5 years of age, was predictive of fat mass at 9 years of age among UK children [14]. In addition, the preferred consumption of fast foods itself has repeatedly been related to an unfavorable body composition among children and adolescents [43,44,45,46] even though it is possible that associations with measures of obesity risk may partly reflect relations to the associated behaviors rather than the preferred consumption of these foods [47,48]. The fact that no predictive fast food pattern was identified for the IDEFICS-Germany cohort may, in part, result from differences in the focused inquiry of these foods by the employed FPQs: in KOPS, one-third of the inquired food groups (8 out of 22) referred to fast foods while only four out of 43 items in IDEFICS-Germany addressed fast foods.

Our study did also confirm a protective role of a Mediterranean type pattern identified by PCA, which was prospectively related to lower odds for excess gains in BMI, FMI, and WtHR in the German IDEFICS-Germany cohort. Similar associations have previously been reported by some [49,50,51,52,53] but not all [54,55] pediatric studies that were explicitly investigating the relevance of Mediterranean dietary indices or scores for body composition. Recently, a Mediterranean type diet was observed to be associated with a more favorable BMI, glucose, and lipid profile among children and adolescents presenting with components of the metabolic syndrome than a standard diet [56]. Similarly, evidence from both intervention and prospective cohort studies support benefits of Mediterranean type diets for body composition among adults [57,58,59,60].

Snacking habits have also been suggested as a potential cause contributing to the development of overweight and obesity among children. Evidence for the prospective role of a ‘snacking’ pattern among children is available from two prospective observational studies. In a cohort of 5-year-old to 12-year-old Columbian children, the adherence to this PCA-derived ‘snacking’ pattern was associated with greater increases in BMI, subscapular: triceps skinfold thickness ratio, and waist circumference over a median 2.5 year follow-up [13]. By contrast, a PCA-derived ‘snacking’ pattern in 2-year-old and 9.9-year-old participants of the IDEFICS-multicenter cohort was not related to a two-year BMI change [12]. In line with this, the PCA ‘snack food pattern’ and the ‘change towards a snack pattern’ identified in the IDEFICS-Germany cohort were not related to a higher BMI, FMI, or WtHR. PCA generally yields interpretable patterns, but they may not be relevant for disease outcomes because PCA is purely exploratory, which explains maximal variation in food intake [61]. In addition, PCA patterns generally tend to account for only a small amount of the total variance in food intake in pediatric populations [61,62,63,64,65]. Similarly, in our study, the PCA patterns accounted for only 6.4% to 12.7% and 4.5% to 10.3% of the total variance in food intake in KOPS and IDEFICS-Germany, respectively.

RRR may be more informative since it explicitly derives predictors that explain maximal variation in response variables [40,66]. In the present study, three of the RRR patterns point towards a specific relevance of combinations of unfavorable carbohydrate sources and (animal) protein sources (RRR patterns ‘fast food’, ‘increase in the consumption of fast foods and starchy carbohydrate foods,’ and ‘decrease in the consumption of protein sources and snack carbohydrates.’ The protein leverage hypothesis proposes that a preference for protein in combination with a decrease in the ratio of protein to carbohydrates and fat in the diet is related to excess energy intake and the risk of obesity [67,68]. The two RRR change patterns in the present study are in line with this since they suggest that either increases in (starchy) carbohydrate sources or decreases in the consumption of protein sources during primary school years were associated with an unfavorable development of body composition. Data from a large animal study conducted by the advocates of the protein leverage hypothesis revealed that a high ratio of protein to carbohydrate rather than total caloric intake was related to lower body fat content but may adversely affect cardio-metabolic health [69]. However, in adults, diets with higher plant and animal protein intake were associated with lower central adiposity [70,71] and favorable cardiometabolic markers [71]. Furthermore, in eight-year-old school children, high protein intake was positively associated with a higher fat-free mass index two years later [72]. The relevance of macronutrient ratios or of the combinations of carbohydrate and protein sources for body composition remains to be elucidated.

Despite some similarities in the identified pattern and their association with body composition development in primary school years as outlined above, the patterns differ substantially between both cohorts. Our analyses do, therefore, not confirm our initial hypothesis that similar detrimental or protective dietary patterns may be identified in the three German pediatric cohorts, i.e., DONALD, KOPS, and IDEFICS-Germany. It is likely that this largely reflects the considerable methodological differences, i.e., the different nutrition assessment methods as well as differences in the examined food groups. Comparison with other pediatric studies is further hampered by differences in the method to identify dietary patterns, e.g., the definition of high factor loadings and the fact that the simplified pattern approach is not universally applied.

To overcome some of the issues of comparability, both the criteria for sample selections and the analytical approach of the present study were similar to those previously applied in the DONALD study [9]. Additional strengths of the present study include its prospective nature, which covered the period of two and 3.6 years of primary school for IDEFICS-Germany and KOPS, respectively, with completion of one FPQ at the beginning and one at the end of the observation period as well as anthropometric examination at both points in time, which allows the analysis of changes in diet and growth. In addition, in both cohorts, FMI and WtHR—i.e., more sensitive measurements of obesity and central adiposity, respectively, were examined in addition to BMI. Nonetheless, the fact that fat mass was estimated by two different algorithms derived from the respective BIA may be seen as a limitation. In addition, changes in body composition after puberty onset are known to differ between early-maturing and late-maturing children of the same age [73]. However, puberty onset was not assessed in the studies. KOPS data stem from 2001 and one can assume that dietary behavior and other lifestyle factors may have changed in the meantime. However, results of examinations conducted in a further cohort after 10 years at school entry (2006–2008) and at the end of the primary school period (4th grade, 2010–2012) indicate that these data are still comparable [74,75]. In addition, our study is limited by the comparably small samples from the KOPS and IDEFICS-Germany cohorts. However, it has been suggested that the ratio of the sample size to the number of food groups may be more important than the absolute number of subjects [76]. We performed a number of statistical tests. Therefore, the chance may be an explanation for our findings. However, for the RRR results in particular consistency of findings across the different body composition outcomes argues against chance findings. Another limitation arises from the fact that the FPQ used in KOPS and IDEFICS-Germany did not inquire the consumption frequencies of the same food groups. Therefore, pooled analyses of the KOPS and IDEFICS-Germany samples were not possible. However, as outlined above, comparative analyses using similar analytic approaches based on the food-group specific data available from different assessment methods are desirable and were conducted.

## 5. Conclusions

The present study confirms an unfavorable impact of fast foods on the development of body composition during primary school years. The role of combinations of unfavorable carbohydrate sources and (animal) protein sources requires further investigation.

## Figures and Tables

**Table 1:** Characteristics of the KOPS and IDEFICS-Germany samples included in the present analyses.

	KOPS		IDEFICS-Germany	
	Median or *n*-Number	P25, P75 or Percentage	Median or *n*-Number	P25, P75 or Percentage
*n* (% female)	372	49.5	298	47.3
Birth and infancy				
Birth year (min–max)	1991–1996		2000–2003	
Gestational age (weeks)	40	39, 40	n/a	
Birth weight < 3500 g, *n* (%)	184	49.5	113	37.9
Appropriate for gestational age, *n* (%)	276	74.2	n/a	
Fully breast fed, *n* (%) ^a^	317	85.2	182	61.1
Smoking during pregnancy, *n* (%)	63	16.9	50	17.2
Family				
Maternal overweight, *n* (%) ^b,c^	104	28.0	116	39.2
Paternal overweight, *n* (%) ^b,c^	161	44.9	165	61.3
Parental overweight, *n* (%) ^b,d^	209	56.2	205	68.8
Parental education, *n* (%) ^e^	217	58.3	149	50.0
Single parenting, *n* (%) ^c,f^	57	15.5	33	11.1
Low income, *n* (%) ^c,g^	10	8.3	47	17.2

Numbers are medians (P25, P75) or *n*-numbers (percentages) unless otherwise indicated. ^a^ KOPS: defined as full breastfeeding > 0.5 months. IDEFICS-Germany: defined as full breastfeeding > 1 months vs. no breastfeeding/missing. ^b^ BMI ≥ 25 kg/m^2^. ^c^ Missing data KOPS: paternal overweight: *n* = 13. Single parenting: *n* = 4. Low income: *n* = 251. Missing data IDEFICS-Germany: maternal overweight: *n* = 2, paternal overweight: *n* = 29, and low income: *n* = 24. ^d^ At least one parent is overweight. ^e^ At least one parent had ≥12 years of schooling. ^f^ IDEFICS-Germany: Single parenting defined as living with mother or father or 50% of the time with each mother and father. ^g^ KOPS: low or low/medium income level, i.e., <1,000 € per months. IDEFICS-Germany: low income level, i.e., <1,100 € per months.

**Table 2:** Anthropometric characteristics of the KOPS and IDEFICS-Germany samples at the beginning (baseline) and the end (follow-up) of the primary school period.

	Beginning of the Primary School Period		End of the Primary School Period		Mean Difference between End and Beginning of Primary School Period	
	Median or *n*-Number	P25, P75, or Percentage	Median or *n*-Number	P25, P75, or Percentage	Median or *n*-Number	P25, P75, or Percentage
KOPS						
*n* (% female)	372	49.5	372	49.5		
Age	6.2	6.0, 6.5	9.8	9.6, 10.1	3.6	3.5, 3.7
Anthropometry						
BMI (kg/m^2^)	15.41	14.63, 16.31	16.85	15.55, 18.35	1.32	0.58, 2.55
Overweight, *n* (%)^a^	37	10.0	58	15.6		
FMI (kg/m^2^)	3.18	2.59, 3.91	3.17	2.28, 4.31	−0.02	−0.70, 1.00
FFMI (kg/m^2^)	12.31	11.68, 12.91	13.67	13.11, 14.40	1.46	0.92, 1.97
Waist circumference (cm)	54.5	52.3, 57.5	62.0	58.0, 66.0	7.0	4.0, 10.5
Waist-to-height ratio	0.46	0.44, 0.48	0.43	0.42, 0.46	−0.02	−0.04, 0.01
WtHR >0.5^b^, *n* (%)	46	12.4	36	9.7		
IDEFICS-Germany						
*n* (% female)	298	47.3	298	47.3		
Age	6.5	5.6, 6.9	8.5	7.7, 9.0	2.0	2.0, 2.1
Anthropometry						
BMI (kg/m^2^)	15.50	14.60, 16.60	16.15	14.90, 17.80	0.60	0.10, 1.30
Overweight, *n* (%) ^a^	34	11.4	48	16.1		
FMI (kg/m^2^)	4.33	3.68, 5.17	4.42	3.40, 5.68	0.07	−0.44, 0.73
FFMI (kg/m^2^)	11.23	10.58, 11.79	11.85	11.22, 12.36	0.61	0.39, 0.82
Waist circumference (cm)	51.6	49.5, 54.1	56.5	53.3, 60.1	4.8	2.7, 6.9
Waist-to-height ratio	0.43	0.41, 0.45	0.42	0.40, 0.45	−0.01	−0.02, 0.01
WtHR >0.5 ^b^, *n* (%)	12	4.0	16	5.4		

The numbers are medians (P25, P75) or *n*-numbers (percentages). Differences between baseline and follow-up were tested using the Wilcoxon rank sum test for continuous variables and a Chi-square test for categorical variables. BMI, body mass index, FMI, fat-mass index, WtHR, waist-to-height ratio, ^a^ Derived from the age-specific and sex-specific cut-points proposed by the International Obesity Task Force [36]. ^b^ The cut-off >0.5 was proposed by McCarthy et al. [34] as indicated whether the amount of upper body fat accumulation is excessive and a risk to health.

**Table 3:** Food groups included in the PCA and RRR patterns—KOPS sample, *n* = 372.

	**Included Food Groups**	**Factor Loading**	**Explained Variance in the Food Group Intake (%)**
PCA pattern ^a^			
PCA baseline pattern 1 (‘fast food pattern’)	+ Fish sticks	0.62	12.7
	+ Curry-sausage	0.62	
	+ Lasagna	0.57	
	+ Pancakes	0.55	
	+ Potato fritters	0.52	
	+ Pizza	0.51	
	+ Meat balls	0.5	
PCA baseline pattern 2 (‘wholegrain, vegetables & fruits’)	+ Whole-grain bread	0.66	8.1
	+ Vegetables, salad	0.65	
	+ Fruits	0.56	
	+ Cheese, curd, yogurt	0.47	
	+ Muesli	0.43	
PCA change pattern 1 (‘increase in the consumption of fast food’)	+Δ Meat balls	0.61	9.4
	+Δ Fish sticks	0.57	
	+Δ Lasagna	0.54	
	+Δ Pizza	0.52	
	+Δ Pancakes	0.48	
	+Δ Curry-sausage	0.44	
	+Δ Potato fritters	0.43	
PCA change pattern 2 (‘increase in the consumption of vegetables and fruits’)	+Δ Vegetables, salad	0.61	6.8
	+Δ Fruits	0.57	
	+Δ Meat	0.47	
	+Δ Potatoes	0.44	
PCA change pattern 3 (‘change towards unhealthy carbohydrates’)	+Δ White bread	0.64	6.4
	+Δ Savory bakery goods	0.49	
	−Δ Whole-grain bread	−0.51	
	**Included Food Groups**	**Factor Loading**	**Explained Variance in Response Variables ^b^ (%)**
RRR pattern ^a^			
RRR baseline pattern (‘fast food pattern’)	− Whole-grain bread	−0.32	Changes in BMI: 9.1 Changes in FMI: 5.5 Changes in WtHR: 7.9 Total variance: 7.5
	− Cheese, curd, yogurt	−0.32	
	+ Lemonade	0.55	
	+ Children’s yogurt	0.41	
	+ Potato fritters	0.27	
	+ Meat balls	0.24	
	+ Meat	0.21	
RRR change pattern (‘increase in the consumption of fast foods and starchy carbohydrate foods’)	+Δ Fish sticks	0.49	Changes in BMI: 7.7 Changes in FMI: 5.9 Changes in WtHR: 8.6 Total variance: 7.4
	+Δ Whole-grain bread	0.26	
	+Δ Pizza	0.21	
	+Δ Potatoes	0.2	
	−Δ Vegetables	−0.44	
	−Δ Lemonade	−0.33	
	−Δ Sweets	−0.28	
	−Δ Chocolate spread	−0.21	
	−Δ Meat balls	−0.21	

+, positive loading, −, negative loading, Δ, change, BMI, body mass index, FMI, fat-mass index, WtHR, waist-to-height ratio. ^a^ PCA patterns consider food groups with factor loadings ≥|0.4|, RRR patterns consider food groups with factor loadings ≥|0.2|. ^b^ Changes in response variables between the beginning of the primary school period (baseline) and the end of the primary school period adjusted for the baseline.

**Table 4:** Food groups included in the PCA and RRR patterns—IDEFICS-Germany sample, *n* = 298.

	**Included Food Groups**	**Factor Loading**	**Explained Variance in Food Group Intake (%)**
PCA pattern^a^			
PCA baseline pattern 1 (‘snack pattern’)	+ Sweet snacks (biscuits, packaged cakes, pastries, puddings)	0.57	10.3
	+ Potatoes (fried, croquettes)	0.52	
	+ Ketchup and similar	0.51	
	+ Savory snacks (Crisps, corn crisps, popcorn)	0.51	
	+ Sweetened drinks	0.45	
	+ Chocolate, candy bars	0.44	
	+ Candies, loose candies, marshmallows	0.44	
	+ Ice cream, milk, or fruit-based bars	0.43	
PCA baseline pattern 2 (‘Mediterranean type pattern’)	+ Plain unsweetened yogurt or kefir	0.66	6.5
	+ Dish of milled cereals	0.57	
	+ Nuts, seeds, dried fruits	0.56	
	+ Pizza as main dish	0.45	
	+ Cheese (sliced and spreadable)	0.44	
	+ Fresh meat, not fried	0.44	
	+ Plain unsweetened milk	0.42	
	+ Fresh fruits with added sugar	0.41	
	+ Water	0.41	
	+ Pasta, noodles, rice	0.41	
PCA change pattern 1 (‘change towards a Mediterranean type pattern’)	+Δ Nuts, seeds, dried fruits	0.6	8.9
	+Δ Pasta, noodles, rice	0.56	
	+Δ Fresh meat, not fried	0.52	
	+Δ Pizza as main dish	0.49	
	+Δ Dish of milled cereals	0.48	
	+Δ Sweet yogurt, fermented milk beverages	0.43	
	+Δ Fried meat	0.41	
PCA change pattern 2 (‘change towards a traditional type pattern’)	+Δ Cooked vegetables, potatoes, beans, and legumes	0.54	4.8
	+Δ Sweetened drinks	0.49	
	+Δ Butter, margarine on bread	0.48	
	+Δ Fresh fruits without added sugar	0.47	
PCA change pattern 3 (‘change towards a snack pattern’)	+Δ Sweet snacks (biscuits, packaged cakes, pastries, puddings)	0.65	4.5
	+Δ Candies, loose candies, marshmallows	0.58	
	+Δ Ice cream, milk, or fruit-based bars	0.52	
	+Δ Savory snacks (Crisps, corn crisps, popcorn)	0.51	
	+Δ Chocolate, candy bars	0.46	
	**Included Food Groups**	**Factor Loading**	**Explained Variance in Response Variables b (%)**
RRR pattern ^a^			
RRR baseline pattern (‘Nuts, meat, and pizza pattern’)	+ Nuts, seeds, dried fruits	0.37	Changes in BMI: 11.5 Changes in FMI: 11.9 Changes in WtHR: 12.3 Total variance: 11.9
	+ Fresh meat, not fried	0.36	
	+ Pizza as main dish	0.3	
	+ Plain unsweetened yogurt or kefir	0.23	
	+ Jam, honey	0.22	
	+ Savory pastries, fritters	0.23	
	+ Dish of milled cereals	0.24	
	− Cooked vegetables, potatoes, beans, and legumes	−0.29	
	− Breakfast cereals, muesli, sweetened	−0.23	
RRR change pattern (‘decrease in the consumption of protein sources and snack carbohydrates’)	+Δ Reduced-fat products on bread	0.24	Changes in BMI: 14.0 Changes in FMI: 14.3 Changes in WtHR: 12.0 Total variance: 13.5
	−Δ Fresh meat, not fried	−0.36	
	−Δ Savory pastries, fritters	−0.33	
	−Δ Fried or scrambled eggs	−0.29	
	−Δ Sweetened drinks	−0.27	
	−Δ Nuts, seeds, dried fruits	−0.3	
	−Δ Dish of milled cereals	−0.24	

+, positive loading, −, negative loading, Δ, change, BMI, body mass index, FMI, fat-mass index, WtHR, waist-to-height ratio. ^a^ PCA patterns consider food groups with factor loadings ≥|0.4|, RRR patterns consider food groups with factor loadings ≥|0.2|. ^b^ Changes in response variables between the beginning of the primary school period (baseline) and the end of the primary school period adjusted for baseline.

**Table 5:** Odds for excess gains in BMI, fat-mass index (FMI), or waist-to-height ratio (WtHR) ^a^ during the primary school period, according to tertiles of adherence to the PCA patterns—KOPS sample (*n* = 372).

	**PCA Baseline Pattern 1 (‘Fast Food Pattern’)**								**PCA Change Pattern 1 (‘Increase in the Consumption of Fast Food’)**							
	**T1**	**T2**		**T3**		**Continuous ^b^**		***p*_trend_^c^**	**T1**	**T2**		**T3**		**Continuous ^b^**		***p*_trend_^c^**
		**OR**	**95% CI**	**OR**	**95% CI**	**OR**	**95% CI**			**OR**	**95% CI**	**OR**	**95% CI**	**OR**	**95% CI**	
BMI																
Model A	1	0.89	0.50, 1.60	1.26	0.72, 2.21	1.03	0.98, 1.09	0.2768	1	0.91	0.50, 1.65	1.39	0.79, 2.46	1.04	0.99, 1.10	0.1478
Model B		0.81	0.44, 1.49	1.00	0.55, 1.80	1.01	0.95, 1.07	0.8457	1	0.95	0.51, 1.75	1.49	0.83, 2.68	1.05	0.99, 1.10	0.1133
FMI																
Model A	1	0.79	0.44, 1.40	0.98	0.56, 1.73	1.02	0.97, 1.08	0.4590	1	1.10	0.60, 2.01	1.73	0.97, 3.07	1.05	1.00, 1.11	0.0538
Model B	1	0.67	0.37, 1.24	0.73	0.41, 1.33	0.99	0.94, 1.06	0.8556	1	1.19	0.64, 2.21	1.86	1.03, 3.38	1.06	1.00, 1.12	0.0411
WtHR																
Model A	1	0.59	0.32, 1.07	1.11	0.64, 1.93	1.03	0.97, 1.09	0.3318	1	0.76	0.42, 1.38	1.28	0.73, 2.23	1.02	0.97, 1.08	0.4498
Model B	1	0.54	0.29, 1.01	0.88	0.49, 1.57	1.00	0.95, 1.06	0.9329	1	0.79	0.43, 1.46	1.35	0.76, 2.40	1.02	0.97, 1.08	0.3665
	**PCA Baseline Pattern 2 (‘Wholegrain, Vegetables & Fruits’)**								**PCA Change Pattern 2 (‘Increase in the Consumption of Vegetables and Fruits’)**							
	**T1**	**T2**		**T3**		**Continuous ^b^**		***p*_trend_^c^**	**T1**	**T2**		**T3**		**Continuous ^b^**		***p*_trend_^c^**
		**OR**	**95% CI**	**OR**	**95% CI**	**OR**	**95% CI**			**OR**	**95% CI**	**OR**	**95% CI**	**OR**	**95% CI**	
BMI																
Model A	1	0.94	0.54, 1.64	0.60	0.33, 1.09	0.95	0.88, 1.03	0.2316	1	1.01	0.57, 1.79	0.87	0.49, 1.55	1.00	0.92, 1.09	0.9687
Model B		1.10	0.62, 1.95	0.77	0.41, 1.43	1.00	0.91, 1.08	0.9079	1	1.03	0.57, 1.86	0.76	0.42, 1.39	0.98	0.90, 1.08	0.7236
FMI																
Model A	1	1.20	0.69, 2.09	0.68	0.37, 1.25	0.96	0.89, 1.04	0.2859	1	0.85	0.47, 1.52	1.03	0.59, 1.82	1.02	0.93, 1.11	0.7210
Model B	1	1.42	0.80, 2.53	0.89	0.47, 1.67	1.00	0.92, 1.09	0.9971	1	0.86	0.47, 1.57	0.92	0.51, 1.65	1.00	0.91, 1.09	0.9852
WtHR																
Model A	1	0.87	0.50, 1.51	0.58	0.32, 1.05	0.96	0.89, 1.04	0.3049	1	0.65	0.36. 1.17	0.91	0.52, 1.59	0.98	0.90, 1.07	0.6387
Model B	1	0.97	0.55, 1.72	0.72	0.39, 1.34	1.00	0.92, 1.09	0.9676	1	0.61	0.33, 1.13	0.80	0.45, 1.43	0.96	0.88, 1.05	0.3628
									**PCA Change Pattern 3 (‘Change towards Unhealthy Carbohydrates’)**							
									**T1**	**T2**		**T3**		**Continuous ^b^**		***p*_trend_^c^**
										**OR**	**95% CI**	**OR**	**95% CI**	**OR**	**95% CI**	
BMI																
Model A									1	0.83	0.47, 1.46	0.80	0.45, 1.42	0.97	0.87, 1.08	0.5265
Model B									1	0.89	0.50, 1.61	0.90	0.50, 1.63	0.99	0.88, 1.10	0.8046
FMI																
Model A									1	0.69	0.39, 1.24	0.84	0.48, 1.47	0.95	0.85, 1.06	0.3516
Model B									1	0.75	0.41, 1.35	0.96	0.53, 1.72	0.97	0.87, 1.09	0.6091
WtHR																
Model A									1	0.87	0.49, 1.52	0.67	0.37, 1.20	0.91	0.81, 1.01	0.0836
Model B									1	0.92	0.52, 1.64	0.71	0.39, 1.30	0.92	0.82, 1.03	0.1262

Values are odds ratios (95% confidence intervals) presented in tertiles of adherence to the respective dietary pattern. ^a^ Change in body composition was adjusted for the respective baseline value using the residual method. Odds refer to excess gains in BMI, FMI, or WtHR’ defined as gains >75th percentile. ^b^ Continuous, i.e., per unit of pattern score. ^c^ Based on logistic regression models with dietary pattern scores as a continuous variable. Model A: Unadjusted. Model B: Adjusted for parental overweight (BMI ≥ 25 kg/m^2^, yes/no), parental education (≥12 years of schooling, yes/no), and physical activity (very low, low, middle, high).

**Table 6:** Odds for excess gains in BMI, fat-mass index (FMI), or waist-to-height ratio (WtHR) ^a^ during the primary school period according to tertiles of adherence to the PCA patterns—IDEFICS-Germany sample (*n* = 298).

	**PCA Baseline Pattern 1 (‘Snack Pattern’)**								**PCA Change Pattern 1 (‘Increase in the Consumption of Fast Food’)**							
	**T1**	**T2**		**T3**		**Continuous ^b^**		***p*_trend_^c^**	**T1**	**T2**		**T3**		**Continuous ^b^**		***p*_trend_^c^**
		**OR**	**95% CI**	**OR**	**95% CI**	**OR**	**95% CI**			**OR**	**95% CI**	**OR**	**95% CI**	**OR**	**95% CI**	
BMI																
Model A	1	0.69	0.37, 1.29	0.52	0.27, 1.00	0.95	0.88, 1.02	0.1537	1	1.15	0.62, 2.15	0.67	0.34, 1.30	0.93	0.87, 1.00	0.0395
Model B		0.67	0.35, 1.26	0.47	0.23, 0.91	0.94	0.87, 1.01	0.0810	1	1.12	0.60, 2.11	0.65	0.33, 1.30	0.93	0.87, 0.99	0.0436
FMI																
Model A	1	1.10	0.58, 2.07	0.85	0.44, 1.63	0.99	0.92, 1.06	0.8102	1	1.21	0.65, 2.27	0.75	0.38, 1.46	0.93	0.87, 0.99	0.0454
Model B	1	1.06	0.55, 2.03	0.78	0.39, 1.52	0.98	0.91, 1.05	0.6013	1	1.05	0.55, 1.99	0.79	0.40, 1.56	0.94	0.87, 0.99	0.0490
WtHR																
Model A	1	1.29	0.68, 2.45	1.00	0.52, 1.94	0.99	0.92, 1.06	0.8259	1	1.04	0.55, 1.95	0.76	0.39, 1.46	0.93	0.87, 0.99	0.0409
Model B	1	1.18	0.62, 2.28	0.95	0.48, 1.87	0.98	0.92, 1.05	0.6481	1	1.04	0.55, 1.99	0.79	0.40, 1.56	0.93	0.87, 0.99	0.0490
	**PCA Baseline Pattern 2 (‘Mediterranean Type Pattern’)**								**PCA Change Pattern 2 (‘Change towards a Traditional Type Pattern’)**							
	**T1**	**T2**		**T3**		**Continuous ^b^**		***p*_trend_^c^**	**T1**	**T2**		**T3**		**Continuous ^b^**		***p*_trend_^c^**
		**OR**	**95% CI**	**OR**	**95% CI**	**OR**	**95% CI**			**OR**	**95% CI**	**OR**	**95% CI**	**OR**	**95% CI**	
BMI																
Model A	1	0.65	0.33, 1.25	0.92	0.49. 1.71	1.04	0.99, 1.09	0.1456	1	1.04	0.55, 1.99	1.06	0.55, 2.02	1.02	0.92, 1.13	0.7179
Model B		0.65	0.33, 1.27	0.92	0.47, 1.79	1.04	0.99, 1.11	0.1403	1	1.02	0.52, 1.98	1.06	0.54, 2.07	1.02	0.92, 1.14	0.6587
FMI																
Model A	1	0.96	0.51, 1.82	0.86	0.45, 1.64	1.03	0.98, 1.09	0.1957	1	1.10	0.58, 2.07	0.85	0.44, 1.63	0.99	0.89, 1.10	0.8205
Model B	1	1.00	0.52, 1.92	0.91	0.46, 1.81	1.05	0.99, 1.12	0.1073	1	1.02	0.53, 1.96	0.83	0.42, 1.63	0.99)	0.89, 1.10	0.8780
WtHR																
Model A	1	0.62	0.32, 1.19	0.83	0.44, 1.55	1.04	0.98, 1.10	0.2349	1	0.75	0.39, 1.44	0.90	0.48, 1.70	0.95	00.86, 1.05	0.3343
Model B	1	0.60	0.31, 1.17	0.86	0.44, 1.67	1.04	0.98, 1.11	0.1764	1	0.63	0.32, 1.24	0.77	0.40, 1.50	0.94	0.85, 1.04	0.2324
									**PCA Change Pattern 3 (‘Change towards a Snack Pattern’)**							
									**T1**	**T2**		**T3**		**Continuous ^b^**		***p*_trend_^c^**
										**OR**	**95% CI**	**OR**	**95% CI**	**OR**	**95% CI**	
BMI																
Model A									1	1.53	0.80, 2.99	1.41	0.73, 2.76	1.01	0.93, 1.10	0.7551
Model B									1	1.60	0.83, 3.15	1.46	0.75, 2.87	1.02	0.94, 1.11	0.6363
FMI																
Model A									1	1.24	0.64, 2.41	1.47	0.77, 2.83	0.98	0.90, 1.06	0.6550
Model B									1	1.27	0.65, 2.50	1.49	0.78, 2.90	0.99	0.91, 1.08	0.8546
WtHR																
Model A									1	1.11	0.57, 2.18	1.62	0.85, 3.11	1.03	0.95, 1.12	0.5336
Model B									1	1.17	0.59, 2.33	1.64	0.86, 3.19	1.03	0.95, 1.12	0.4342

Values are odds ratios (95% confidence intervals) presented in tertiles of adherence to the respective dietary pattern. ^a^ Change in body composition was adjusted for the respective baseline value using the residual method. Odds refer to excess gains in BMI, FMI, or WtHR defined as gains >75th percentile. ^b^ Continuous, i.e., per unit of pattern score. ^c^ Based on logistic regression models with dietary pattern scores as a continuous variable. Model A: Unadjusted. Model B: Adjusted for parental overweight (BMI ≥ 25 kg/m^2^, yes/no), smoking during pregnancy (yes/no), and migration background (born in Germany, yes/no) in models for BMI and FMI additional adjustment for low income (<1.100 € per months, (no/yes/unknown): 8% of values were imputed using the missing indicator method resulting in a coding of 0, 1, and 2, respectively [42]).

## Supplementary Materials

The following are available online at http://www.mdpi.com/2072-6643/10/10/1442/s1, Table S1: Food groups assessed by the food propensity questionnaire (FPQ) in KOPS at baseline (*n* = 372). Table S2: Food groups assessed in IDEFICS-Germany by the Children’s Eating Habits Questionnaire (CEHQ) at baseline (*n* = 298).
