# Supplementary material for: Dietary Patterns in Primary School are of Prospective Relevance for the Development of Body Composition in Two German Pediatric Populations
Source: Nutrients. 2018 Oct 5;10(10):1442. doi: 10.3390/nu10101442 (PMC6213904; doi:10.3390/nu10101442)
Supplement: Supplementary file 1 [file nutrients-10-01442-s001.pdf]

Table S1. Food groups assessed by the food propensity questionnaire (FPQ) in KOPS at baseline (n=372).

Food group	Median[a]	P25	P75
Whole-grain bread	2	2	3
White bread	2	2	3
Cheese, curd, yogurt	2	2	3
Fruits	3	2	3
Vegetables, salad	2	2	3
Sweets	2	2	3
Savory bakery goods	1	0	1
Lemonade	0	0	1
Meat	2	2	3
Chocolate spread	1	0	2
Jam, Honey	1.5	1	2
Cornflakes	2	1	2
Muesli	1	0	2
Children's yogurt	1	0	2
Pasta	1	1	2
Potatoes	2	2	2
French fries	0	0	1
Lasagna	0	0	0
Fish fingers	0	0	1
Pizza	0	0	1
Curry-sausage	0	0	0
Meat balls	0	0	1
Pancakes	0	0	1
Potato fritters	0	0	0

P, percentile; [a] Median food group consumption frequency.

Table S2. Food groups assessed in IDEFICS-Germany by the Children's Eating Habits Questionnaire (CEHQ) at baseline (n=298).

Food group	Median[a]	P25	P75
Cooked vegetables, potatoes and beans, and legumes	4	3	5
Raw vegetables	3	2	4
Potatoes (fried, croquettes)	1	1	2
Fresh fruits without added sugar	4	3	5
Fresh fruits with added sugar	1	1	2
Water	5	5	5
Fruit juices	3	2	5
Sweetened drinks	1	1	3
Diet soft drinks	1	1	2
Plain unsweetened milk	4	3	4
Sweetened milk	2	1	3
Cheese (sliced and spreadable)	4	3	5
Plain unsweetened yogurt or kefir	1	1	2
Sweet yogurt, fermented milk beverages	3	2	3
Fresh fish, not fried	1	1	2
Fried fish, fish fingers	2	1	2
Cured meat and sausages	3	2	4
Fresh meat, not fried	2	1	2
Fried meat	2	2	2
Fried or scrambled eggs	1	1	2
Boiled or poached eggs	1	1	2
Mayonnaise and similar	1	1	1.5
Meat replacement products	1	1	1
Honey, jam	2	1	3
Chocolate or nut-based spreads on bread	2	1	3
Butter, margarine on bread	4	1	5
Low-fat butter, margarine on bread	1	1	4
Ketchup and similar	2	1	2
Breakfast cereals and muesli, sweetened	2	1	3
Breakfast cereals and muesli, unsweetened	1	1	2
Bread, white	3	2	4
Bread, wholemeal	3	2	4
Pasta, noodles, rice	2	2	2
Dish of milled cereals	1	1	2
Pizza as main dish	1	1	1
Hamburger, hot dog, kebab, falafel	1	1	1
Nuts, seeds, dried fruits	1	1	2
Savory snacks (Crisps, corn crisps, popcorn)	1	1	2
Savory pastries, fritters	1	1	1
Chocolate, candy bars	2	1	3
Candies, loose candies, marshmallow	3	2	3
Sweet snacks (biscuits, packaged cakes, pastries, puddings)	2	2	3
Ice cream, milk or fruit based bars	2	1	2

P, percentile; [a] Median food group consumption frequency.
